# Glucose Homeostasis Is Not Affected in a Murine Model of Parkinson’s Disease Induced by 6-OHDA

**DOI:** 10.3389/fnins.2018.01020

**Published:** 2019-01-09

**Authors:** Felipe Azevedo Gomes, Rafael Appel Flores, Maciel Alencar Bruxel, Flávia Natividade da Silva, Eduardo Luiz Gasnhar Moreira, Daniel Breseghello Zoccal, Rui Daniel Prediger, Alex Rafacho

**Affiliations:** ^1^Postgraduate Program in Pharmacology, Federal University of Santa Catarina, Florianópolis, Brazil; ^2^Multicenter Postgraduate Program in Physiological Sciences, Federal University of Santa Catarina, Florianópolis, Brazil; ^3^Department of Physiological Sciences, Center of Biological Sciences, Federal University of Santa Catarina, Florianópolis, Brazil; ^4^Department of Physiology and Pathology, School of Dentistry, São Paulo State University, Araraquara, Brazil

**Keywords:** glucocorticoid, glycemia, lipids, liver, pancreatic islets, Parkinson’s disease

## Abstract

There is a mutual relationship between metabolic and neurodegenerative diseases. However, the causal relationship in this crosstalk is unclear and whether Parkinson’s disease (PD) causes a posterior impact on metabolism remains unknown. Considering that, this study aimed to evaluate the appearance of possible changes in metabolic homeostasis due to 6-hydroxydopamine (6-OHDA) administration, a neurotoxin that damage dopaminergic neurons leading to motor impairments that resemble the ones observed in PD. For this, male Wistar rats received bilateral 6-OHDA administration in the dorsolateral striatum, and the motor and metabolic outcomes were assessed at 7, 21, or 35 days post-surgical procedure. Dexamethasone, a diabetogenic glucocorticoid (GC), was intraperitoneally administered in the last 6 days to challenge the metabolism and reveal possible metabolic vulnerabilities caused by 6-OHDA. Controls received only vehicles. The 6-OHDA-treated rats displayed a significant decrease in locomotor activity, exploratory behavior, and motor coordination 7 and 35 days after neurotoxin administration. These motor impairments paralleled with no significant alteration in body mass, food intake, glucose tolerance, insulin sensitivity, and biochemical parameters (plasma insulin, triacylglycerol, and total cholesterol levels) until the end of the experimental protocol on days 35–38 post-6-OHDA administration. Moreover, hepatic glycogen and fat content, as well as the endocrine pancreas mass, were not altered in rats treated with 6-OHDA at the day of euthanasia (38th day after neurotoxin administration). None of the diabetogenic effects caused by dexamethasone were exacerbated in rats previously treated with 6-OHDA. Thus, we conclude that bilateral 6-OHDA administration in the striatum causes motor deficits in rats with no impact on glucose and lipid homeostasis and does not exacerbate the adverse effects caused by excess GC. These observations indicate that neurodegeneration of dopaminergic circuits in the 6-OHDA rats does not affect the metabolic outcomes.

## Introduction

The first description of Parkinson’s disease (PD) was made by James Parkinson in the early nineteenth century (Pearce, 1989). It is considered the second most prevalent neurodegenerative disease in the world, affecting approximately 1% of the world population over 60 years (Schapira, 2009). The disease is characterized by a slow and progressive loss of dopaminergic neurons from the substantia nigra resulting in a gradual loss of motor functions (Stern et al., 2012). At the diagnosis period, approximately 40% of the dopaminergic neurons are intact and the presence of dopamine in the striatum is restricted to approximately 20–30% of the normal condition (Riederer and Wuketich, 1976). The primary etiology of the disease is not yet well known. However, epidemiological studies associate the etiology with several risk factors such as neuronal aging, genetic and environmental factors, viruses and inadequate eating habits (Marttila et al., 1977; Gorell et al., 1997; Mattson, 2003; Steenland et al., 2006; Goldman, 2010).

A classic animal model of PD is obtained through the neurotoxin 6-hydroxydopamine (6-OHDA), a substance that does not cross the blood-brain barrier and therefore requires its administration to be performed directly on the substantia nigra *pars compacta* (SNpc) or on the striatum (Jackson-Lewis et al., 2012). As demonstrated for the first time by Ungerstedt (1968), an intranigral injection of 6-OHDA in rats rapidly eliminates about 60% of tyrosine hydroxylase (TH)-positive neurons in this brain area, with the subsequent loss of TH-positive terminals in the striatum (Blandini et al., 2008). However, when administered at the striatum, the neurotoxin promotes a slower and progressive degeneration and subsequent loss of neurons in the SNpc, which also occurs in PD context (Sauer and Oertel, 1994; Przedborski et al., 1995; Lee et al., 1996).

Several studies have suggested an association between metabolic-related diseases (i.e., diabetes *mellitus*) and neurodegenerative diseases. Some authors have suggested that diabetes *mellitus* type 2 (T2DM) is one of the risk factors for the development of neurodegenerative diseases such as PD (Hu et al., 2007; Klimek et al., 2015; Pagano et al., 2018). In addition, a higher prevalence of T2DM was reported in patients with PD (Pressley et al., 2003). Although there are some associations between metabolic outcomes and PD, or vice versa, an observational study reports that the prevalence of T2DM in patients with PD is similar to the prevalence of individuals without PD (Becker et al., 2008). Scigliano et al. (2006) reported a lower prevalence of DM2 in patients diagnosed with PD; whereas no differences were found in the risk of developing PD between eutrophic patients and diabetic patients (Simon et al., 2007). However, it is known that PD can damage several brain areas and may affect regions involved in metabolic control such as the hypothalamus (Dayan et al., 2018).

The pathophysiological process of PD is complex since it may involve environmental, genetic and inflammatory risk factors that are not yet well understood. In addition, remains unknown whether the brain damage observed in PD can negatively affect peripheral metabolic aspects such as glycemia, lipidemia, glucose tolerance, and peripheral insulin sensitivity. Thus, there is no definition of which the causal factor is; whether the metabolic dysfunctions favor the development of PD or, if the contrary is true, or if both conditions can occur. Thus, due to the high prevalence of metabolic diseases and PD in elderly populations and the lack of knowledge about the relationship between PD and metabolic outcomes, we aimed to investigate the possible impact of murine model of motor dysfunctions caused by 6-OHDA on glycemic and lipid homeostasis. We also challenged these rats with dexamethasone, a diabetogenic drug, to verify how much these 6-OHDA-treated rats are vulnerable or not to glucocorticoid (GC) adverse effects. Our hypothesis is that rats with motor deficits caused by 6-OHDA administration will exhibit deterioration of glucose tolerance and insulin sensitivity and that these changes will be exacerbated when GCs were administered. Overall, we demonstrated that motor impairments caused by 6-OHDA treatment did not parallel with peripheral metabolic parameters either predispose rats to the diabetogenic GC effects.

## Materials and Methods

### Ethics Statement

The experimental protocol was approved by the Federal University of Santa Catarina Committee for Ethics in Animal Experimentation (Approval ID: 4468101116) in accordance with the Brazilian National Council for Animal Experimentation Control (CONCEA).

### Animals

Thirty-six adults male Wistar rats (starting weight 280–350 g) were housed in a temperature and humidity-controlled environment and kept on a 12-h light–dark cycle (lights on 06:00 – 18:00 h). All rats had *ad libitum* access to food (commercial standard chow for rats, Nuvilab CR-1; Nuvital, Brazil) and filtered tap water. The animals were supplied by the Federal University of Santa Catarina’s Animal Breeding Center.

### Experimental Design and Groups

**FIGURE 1:**
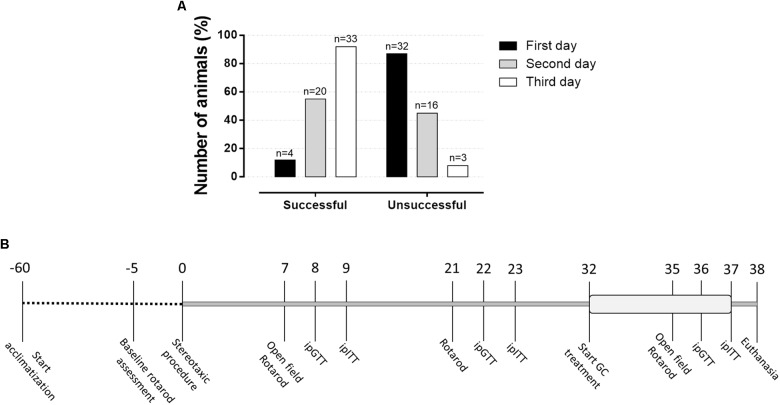
Percentage of animals responsive to the rotarod basal motor protocol and experimental design. **(A)** This test was applied before to initiate the surgeries to identify those rats able to follow in the study. Around 12% of the animals responded to the rotarod test in the first attempt, which achieves approximately 91% of success on the third attempt day. **(B)** The number above line means days along experimental protocol with the respective experimental approaches applied in the study.

Animals were acclimatized for a period of 8 weeks before being randomly assigned in two groups, followed by a new distribution in four experimental groups, as will be described in detail. Animals were submitted to a baseline rotarod assessment protocol and those who were successful in this test were included in the study and underwent stereotaxic surgery. On the first day, only 12% responded adequately to the rotarod test (Figure 1A). The rate of successful increased in the second test day and, on day 3, approximately 91% successfully achieve the inclusion criteria on the rotarod test. Approximately 8% did not adapt to the test and therefore did not follow to the surgical procedure. As shown in Figure 1B, rats were submitted to the open field test on the 7th and 35th days after the surgery and to a second test, rotarod test, on the 7th, 21st, and 35th postoperative days. The intraperitoneal (i.p.) glucose tolerance test (ipGTT) was performed on the 8th, 22nd, and 36th days of the experimental protocol, while the i.p. insulin tolerance test (ipITT) was applied on the 9th, 23rd, and 37th postoperative days. The treatment with dexamethasone started on the 32nd day, which lasted until the 37th day of the experimental protocol (6 days). On day 38, the animals were euthanized, and plasma and organs were collected for further biochemical analysis as described in the next sections.

Groups were defined as follow: initially, rats were randomly allocated in two experimental groups: (1) Control group (Control) (*n* = 17): rats treated only with vehicle via the intra-striatal administration (described in the next section), and (2) 6-hydroxydopamine group (6-OHDA) (*n* = 16): rats treated with 6-hydroxydopamine via the intra-striatal administration (described in the next section). Then, half of the animal from the Control and 6-OHDA groups were treated with dexamethasone composing the following groups, respectively: (3) Dexamethasone group (Dexa) (*n* = 9): received i.p. injection of dexamethasone (as described in other section), and (4) 6-hydroxydopamine+dexamethasone group (6-OHDA + Dexa) (*n* = 8): treated as 6-OHDA and Dexa groups. This experimental protocol was performed in two different sets of animals with half of the number of animals on each occasion.

### Surgical Procedure

Before any surgical procedure, rats were anesthetized with ketamine/xylazine [75/10 mg/kg body mass (b.m.), i.p.]. 6-OHDA (20 μg in 3 μL and an infusion rate of 1 μL/min) (Sigma-Aldrich, St. Louis, MO, United States) was injected bilaterally into the dorsolateral striatum (DLS) at the coordinates: AP: +0.2 mm, ML: ±3.5 mm and DV: −4.8 mm to bregma (Paxinos and Watson, 1997), using a Hamilton 10 μL syringe with a 26-gauge needle connected to a 30-gauge cannula, whereas control rats received vehicle (saline solution). Then, the cannula was left untouchable for an additional minute to prevent backflow. This experimental procedure has been used in rats to generate motor impairments that resemble PD motor impairments (Rampersaud et al., 2012; Hegazy et al., 2017).

### Dexamethasone Treatment

We used dexamethasone to generate an *in vivo* metabolic challenging considering the diabetogenic properties of this GC as previously demonstrated by several studies (Giozzet et al., 2008; Angelini et al., 2010; Rafacho et al., 2014). Dexamethasone disodium phosphate (Decadron^®^, Aché, Campinas, SP, Brazil) was daily injected, through i.p. vial, between 07:30 and 08:30 h, at a dose of 1 mg/kg, b.m., for the last 6 consecutive days.

### Motor Tests

#### Open Field

This test was performed under a red-light (12 lux) condition and with controlled sound, temperature, and humidity. Animals were placed in an arena (100 × 100 cm) during 5 min for free exploration. The arena had smooth sidewalls, measuring 40 cm height, formed by odorless white painted metal and the base was made of concrete coated with black contact paper. The square base had 5 × 5 quadrants, each square with 400 cm^2^ (20 × 20 cm). All experiments were recorded (Logitech C270, Lausanne, Switzerland), and the following parameters were analyzed: (1) number of squares crossed by the animal, which represents the locomotor activity; (2) number of rearing behavior, which represents the exploratory activity; (3) number of quadrants crossed peripherally or centrally, which allows identifying anxiety-related behaviors.

#### Rotarod

This test was applied to evaluate the motor coordination and balance of the animals. The equipment was prepared at three speeds: 12, 20, and 24 revolutions per minute (rpm). The change of speed occurred manually and involved shutdown of the equipment for each speed change. Thus, there was a brief interruption of the test between the rotational changes. All animals were exposed individually to the three different speed levels, consecutively. The baseline assessment protocol consisted of three trials in a constant 12 rpm speed. In the end, rats that remained in the rotarod for 1 min were included in the study and were underwent stereotaxic surgery (Figure 1A). This baseline assessment was initiated 5 days before stereotaxic surgery and was extended for three consecutive days (Figure 1B). On the 7th, 21st, and 35th postoperative days, animals were positioned in the rotarod apparatus, aiming to remain up to 2 min in each speed. The residence time (latency to fall) of each rat at each stage was measured with a conventional digital chronometer as an indicator of balance and motor coordination.

### Measurements of *in vivo* Metabolic Parameters

Verification of body mass was performed every 3 days from the beginning of the experimental protocol to the day of euthanasia in a digital electronic balance (TECNAL; Piracicaba, SP, Brazil). Food intake was monitored as described for body mass. The determination of food intake was performed by weighing the remaining chow discounted from the total of that deposited 24 h before and the mass difference represents the daily amount ingested per cage. The average amount of food ingested per animal was obtained by the following formula [(total chow (g) ingested in the cage/number of rats per cage)/individual rat mass (g)] × 100. The results were expressed as grams (g) of food ingested per 100 g of body mass. During the dexamethasone treatment both body mass and food intake were daily measured, but for aesthetic reasons, only the initial and final measures of food intake were used in the line graph.

### I.p. Glucose Tolerance Test (ipGTT)

The i.p. glucose tolerance test (ipGTT) was performed on 8th, 22nd, and 36th days of the experimental protocol in fasted (08 h) rats, with the experiments occurring around 16:00. The rats had the tip of the tail cut (no more than 1 mm) for blood collection. The first drop was discarded, and the second drop was used for the determination of blood glucose (time 0) using a glucometer (Accu-Chek Performa; Roche Diagnostics, GmbH, Mannheim, Germany). Immediately after, a 50% glucose solution pre-warmed at 36°C (2 g/kg, b.m.) was administered by i.p. injection and blood samples were collected from the tail tip at 30, 60, and 120 min for blood glucose measurements as described before (Giozzet et al., 2008; Motta et al., 2015). Area-under-glucose-curve (AUC) was obtained after normalization by the initial value.

### I.p. Insulin Tolerance Test (ipITT)

The i.p. insulin tolerance test (ipITT) was performed on 9th, 23rd, and 37th days of the experimental protocol in fed rats, with the experiments occurring around 16:00. Blood collection at min 0 occurred as for ipGTT. Then, the animals received an i.p. injection of recombinant human insulin pre-warmed at 36°C (Biohulin^®^ 1 IU/kg b.m.). Blood samples were posteriorly collected from the tail tip at 10, 20, and 40 min for blood glucose measurements (Giozzet et al., 2008; Protzek et al., 2016). AUC was obtained from normalized blood glucose values (expressed as % from min 0).

### Euthanasia and Biochemical Analysis

On the 38th day, 24 h after the last dexamethasone administration, animals received a halothane super dosage (1 mL) (Tanohalo^®^, Cristália, Curitiba, PR, Brazil). Blood samples were collected through heart puncture with EDTA-NaF containing syringes (Glistab – Labtest; Lagoa Santa, MG, Brazil) and centrifuged at 400 *g* for 10 min at 21°C (Eppendorf 5810R). The plasma was separated and stored at −80°C until the posterior determination of insulin, total cholesterol, and triacylglycerol, according to manufacturer instructions and previously published data (dos Santos et al., 2014; Motta et al., 2015). Animals were then continuously perfused through the left ventricle with 20 mL of 0.9% NaCl containing 0.1% heparin followed by infusion of 200 mL of 4% paraformaldehyde (pH 7.4, Sigma-Aldrich). Organs of interest were carefully removed and weighed.

### Liver Glycogen and Hepatic Triacylglycerol Content

Hepatic glycogen content was determined by a phenol-based assay according to a previous publication (Giozzet et al., 2008). Determination of hepatic triacylglycerol content was performed according to a previous publication (Gonçalves-Neto et al., 2014). The same hepatic lobe was used for collection of the fragments for all animals.

### Hepatic Morphology

After perfusion, liver fragments (from the same lobe in all animals) were collected and fixed in 10% buffered formalin, pH 7.4, for 24 h, dehydrated and embedded in paraffin. Representative tissue sections (5 μm) were obtained on a rotating microtome (Leica) and placed on glass slides. Afterward, the slides were submitted to the staining procedure of Hematoxylin and Eosin for further morphological evaluation to verify the presence of lipids and glycogen according to Gonçalves-Neto et al. (2014).

### The Relative Mass of the Endocrine Pancreas

After perfusion, pancreas fragments (splenic region) were collected and fixed in 4% paraformaldehyde for 24 h, dehydrated and embedded in paraffin. Representative tissue sections (5 μm) were obtained on a rotating microtome (Leica) and placed on glass slides. Afterward, the slides were submitted to the staining procedure of hematoxylin and eosin and later scanned by the AxioScan automatic slide scanner (ZEISS, Oberkochen, Germany) for quantification of the relative endocrine pancreatic mass and density of pancreatic islets. To obtain the relative mass data, the total area of the pancreas and pancreatic islets were calculated using the ZEN software (ZEISS). The sum of the areas corresponding to the pancreatic islets was divided by the total pancreas area and multiplied by 100 to obtain the percentage of endocrine pancreas area. To obtain the density of pancreatic islets the following relation was used: islet density = (total number of islets in section × 1000) divided by the total area of the pancreas analyzed. The result was expressed by the number of islets per 1000 μm^2^ of pancreas area (Protzek et al., 2014).

### Statistical Analysis

All analyses were performed using GraphPad Prism Version 6.01 software (GraphPad Inc., La Jolla, CA, United States). The results were expressed as the mean ± SD or mean ± SEM for parametric data or median and interquartile ranges for non-parametric data. The symmetry of the data was tested by Kolmogorov–Smirnov, Shapiro–Wilk, and D’Agostino and Pearson omnibus normality tests. It was considered symmetric if approved by at least one of three tests. Analysis of variance (two-way ANOVA) followed by Tukey’s *post hoc* test was used for multiple comparisons of parametric data or Kruskal–Wallis followed by Dunn’s *post hoc* when the variables presented an asymmetric distribution, while the categorical data were evaluated by the Person Chi-square test. Significance was set at *p* < 0.05.

## Results

### Characterization of the 6-OHDA Model

#### Central Administration of 6-OHDA Caused Reduction in Locomotor Activity in the Open Field Test

**FIGURE 2:**
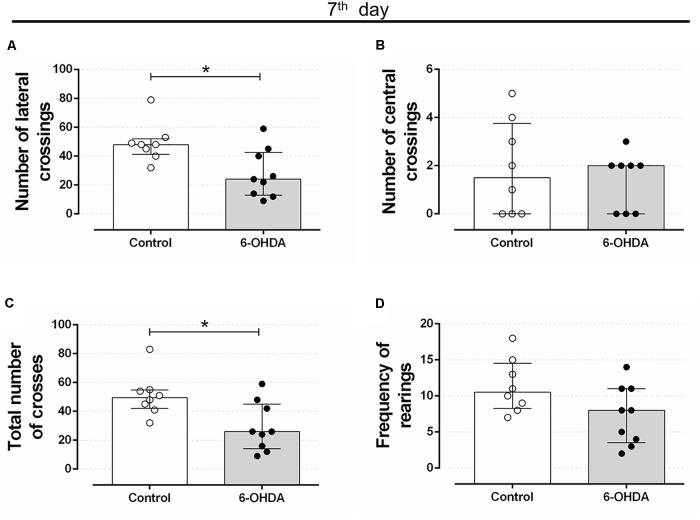
Effect of 6-OHDA on the 7th day of the experimental protocol, in motor parameters evaluated in the open field test. **(A)** number of lateral crossings; **(B)** number of central crossings; **(C)** the total number of crosses (central + lateral); **(D)** average frequency of rearings. Data are median and interquartile range as they are asymmetrically distributed (nonparametric). ^∗^Indicates a significant difference compared to the respective control group using unpaired Mann–Whitney. *n* = 8–9, *p <* 0.05.

Rats treated with 6-OHDA exhibited motor impairment on the 7th day after stereotaxic surgery. The number of lateral and total crosses significantly reduced around 43% in the 6-OHDA group of rats, compared with the Control group (Figures 2A,C, respectively). No significant alterations were observed in the number of central crosses and in the frequency of rearing behavior (Figures 2B,D, respectively).

**FIGURE 3:**
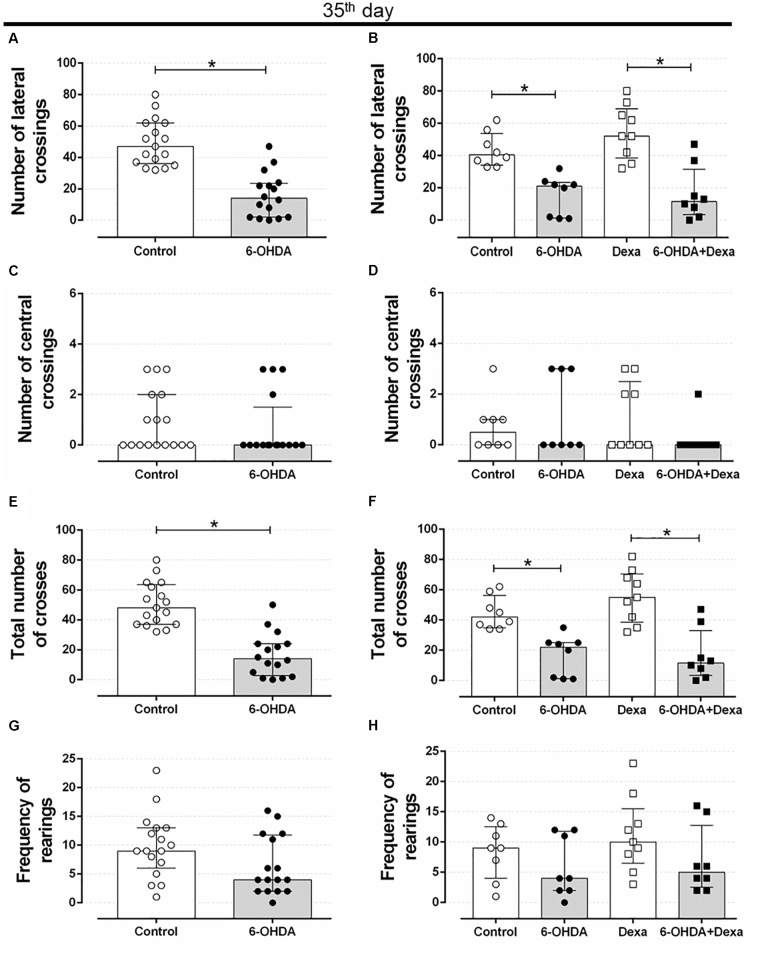
Effect of 6-OHDA on the 35th day of the experimental protocol, in motor parameters evaluated in the open field test. **(A)** number of lateral crosses without distinction of dexamethasone treatment; **(B)** number of lateral crossings; **(C)** number of central crosses without distinction of dexamethasone treatment; **(D)** number of central crossings; **(E)** total number of crosses (central + lateral) without distinction of dexamethasone treatment; **(F)** total number of crosses (central + lateral); **(G)** frequency of rearings without distinction of dexamethasone treatment; **(H)** frequency of rearings. Data are median and interquartile range as they are asymmetrically distributed (nonparametric). ^∗^Indicates a significant difference compared to the respective control groups using unpaired Mann–Whitney in **(A,C,E,G)**, and Kruskal–Wallis with Dunn’s *post hoc* test in **(B,D,F,H)**. *n* = 16–17 in **(A,C,E,G)**, and 8–9 in **(B,D,F,H)**, *p <* 0.05.

Motor impairment addressed on the open field test persisted until the end of experimental protocol (35 days after stereotaxic surgery and on the 3rd day of GC treatment) in rats treated with 6-OHDA, as observed by the significant reduction in the number of lateral crosses (62%) and in the number of total crosses (67%) when compared with the Control group values (Figures 3A,E). This motor impairment was not attenuated or exacerbated by the GC treatment reinforcing the 6-OHDA effect (Figures 3B,F). There were no significant differences in the number of central crosses as well as in the number of rearing behavior in both 6-OHDA and 6-OHDA + Dexa groups compared to their respective Control groups (Figures 3C,D,G,H). Again, treatment with dexamethasone (Dexa and 6-OHDA + Dexa groups) exerted no impact on these parameters.

#### Central Administration of 6-OHDA Caused Reduction in Motor Coordination and Balance Impairments

**FIGURE 4:**
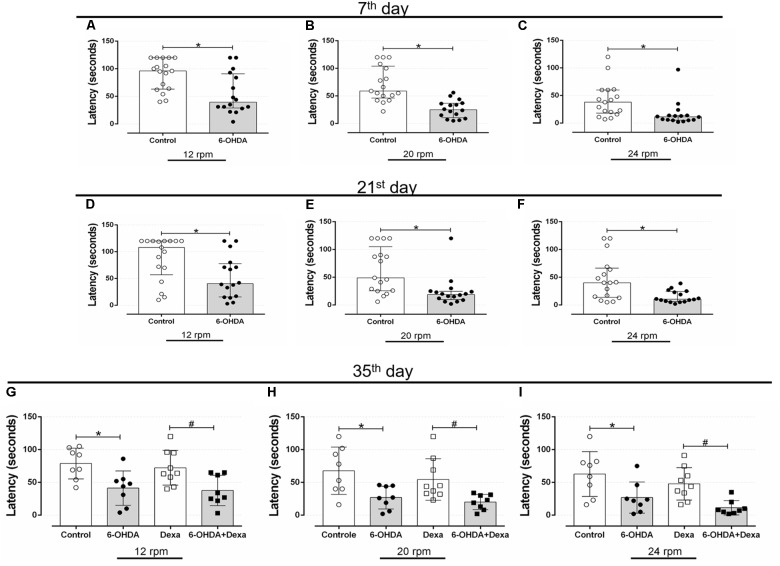
Effect of 6-OHDA injection, on the 7th, 21st, 35th and day of the experimental protocol, in motor parameter evaluated in the rotarod test d. **(A–C)** Latency to fall in seconds at speeds of 12, 20, and 24 revolutions per minute (rpm) at the 7th, **(D–F)** 21st, and **(G–I)** 35th day. Data are median and interquartile range as they are asymmetrically distributed (non-parametric) in **(A–F)**, and mean ± SD in **(G–I)**. ^∗^ and ^#^ Indicates a significant difference compared to the Control and Dexa groups, respectively, using unpaired Mann–Whitney in **(A–F)**, and ordinary two-way ANOVA with Tukey’s *post hoc* test in **(G–I)**. *n* = 16–17 in **(A–F)**, and 8–9 in **(G–I)**, *p <* 0.05.

Rotarod is considered a gold-standard method to evaluate motor coordination in rodents. Bilateral administration of 6-OHDA into the dorsolateral striatum resulted in impairment of the motor coordination of the animals. At the rate of 12 rpm (Figure 4A), the 6-OHDA group had a significant 39% reduction in the latency to rotarod fall on the 7th postoperative day, when compared to the Control group. In addition, the 6-OHDA group also showed a significant reduction of 63 and 60% at the speed of 20 and 24 rpm (Figures 4B,C, respectively).

The latency to fall from the rotarod equipment was very similar at day 21. The reduction in the latency to rotarod fall in the 6-OHDA group was of 40, 62, and 68% at the 12, 20, and 24 rpm speeds, respectively, demonstrating the significant persistence of motor impairment in relation to the Control group (Figures 4D–F).

The same pattern of latency to fall was observed on the 35th day of the experimental protocol (3rd day of GC treatment). The 6-OHDA group remained with a significant reduction of 46% in the rotarod permanence at 12 rpm (Figure 4G), 59% at 20 rpm (Figure 4H), and 54% at 24 rpm (Figure 4I), compared with the Control group. Treatment with dexamethasone did not influence the latency time to fall from rotarod, as observed in Figures 4G–I. Altogether, these findings confirm that bilateral administration of 6-OHDA in male rats is enough to cause motor dysfunctions.

#### Central Administration of 6-OHDA Did Not Impact on Body Mass Gain and Food Intake

**FIGURE 5:**
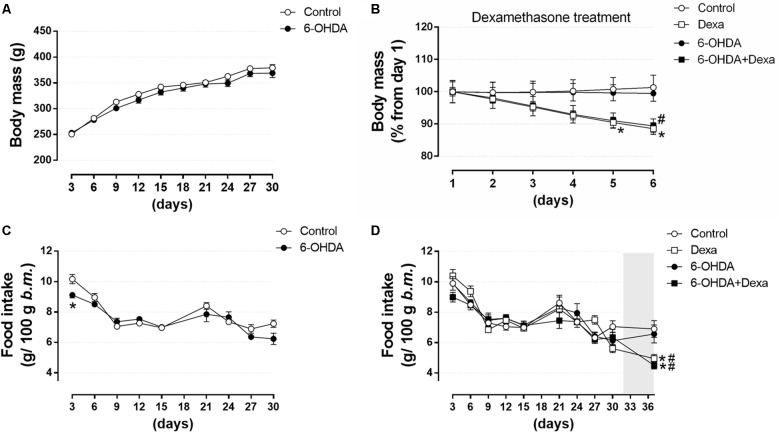
Body mass and food intake over 36 days. **(A)** Body mass of Control and 6-OHDA groups in the first 30 days. **(B)** Normalized body mass (from the 1st day of treatment) of the Control, Dexa, 6-OHDA and 6-OHDA + Dexa groups during the 6 days of dexamethasone treatment. **(C)** Food intake of Control and 6-OHDA groups during the first 30 days. **(D)** Food intake of Control, Dexa, 6-OHDA, and 6-OHDA + Dexa groups during the whole experimental protocol. Data are mean ± SEM (SEM were applied only for aesthetic reasons). ^∗^ and # Indicates a significant difference compared to the Control and 6-OHDA groups, respectively, using unpaired Student’s *t*-test in **(A,C)**, and ordinary two-way ANOVA with repeated measures in **(B,D)**. *n* = 16–17 in **(A,C)**, and 8–9 in **(B,D)**, *p <* 0.05. The light gray color in **(D)** means the period of dexamethasone treatment (six consecutive days).

To verify whether motor dysfunctions resulted from treatment with 6-OHDA was associated to any alteration on murinometric parameters, and whether the treatment with dexamethasone was able to reveal any metabolic vulnerability in those animals, several parameters typical of metabolic studies were addressed. There were no differences in body mass between the Control and 6-OHDA groups during the first 30 days, before the beginning of dexamethasone treatment (Figure 5A). In the 32nd day, the metabolic challenge was initiated by treatment with dexamethasone. From this moment this parameter was analyzed in the four experimental groups. Both the Dexa and 6-OHDA + Dexa groups presented a significant reduction of ∼11% in body mass at the end of the dexamethasone treatment (Δ 44 g and 39 g, respectively) (Figure 5B). Food intake was similar between Control and 6-OHDA groups when analyzed during the first 30 days of the experimental protocol, except for a transient reduction in the consumption of the 6-OHDA group in the first measure of the experimental protocol, when compared to the Control group (Figure 5C). In the 32nd day the metabolic challenge with dexamethasone started, and from this moment on, the parameter was analyzed in the four experimental groups. Both the Dexa and 6-OHDA + Dexa exhibited a significant reduction in food intake of 25 and 32%, respectively, at the end of dexamethasone treatment when compared to their respective controls (Figure 5D).

#### Central Administration of 6-OHDA Did Not Alter Glucose Tolerance and Insulin Sensitivity

**FIGURE 6:**
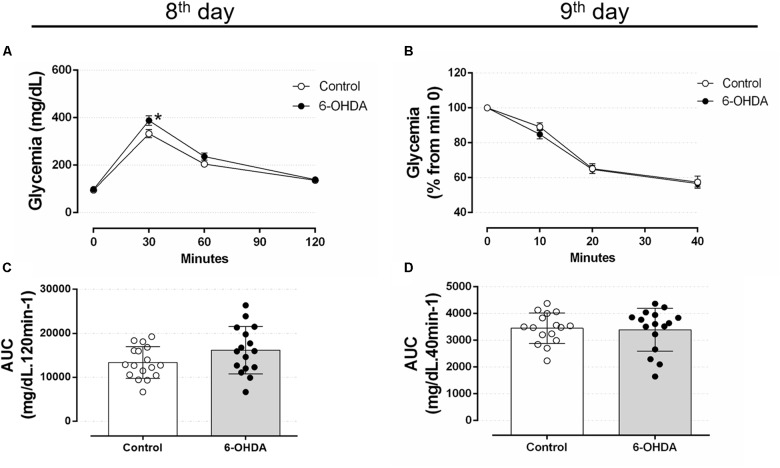
Blood glucose values during the intraperitoneal glucose and insulin tolerance test on the 8th and 9th day of the experimental protocol. **(A)** Blood glucose values during the ipGTT on the 8th day. **(B)** Blood glucose values (normalized from min 0) during the ipITT on the 9th day. **(C)** Area-under-curve during ipGTT and **(D)** during ipITT. Data are mean ± SEM in **(A,B)**, and mean ± SD in **(C,D)** (SEM were applied only for aesthetic reasons). ^∗^Indicates a significant difference compared to the Control using ordinary two-way ANOVA with repeated measures. Unpaired Student’s *t*-test was applied in **(C,D)**. *n* = 16–17, *p <* 0.05.

To verify whether motor impairment caused by 6-OHDA may be associated with glucose homeostasis disturbances, we proceeded with analyzes of metabolic parameters to identify possible alterations associated with the neuromotor outcome. Considering glucose intolerance may anticipate the elevation in blood glucose, we assessed the glucose tolerance on the 8th, 22nd, and 36th days, and the insulin sensitivity on the 9th, 23rd, and 37th days of the experimental protocol. Rats treated with 6-OHDA exhibited a mild increase of blood glucose levels 30 min after an i.p. glucose *bolus* than the Control group (Figure 6A). The blood glucose values were 332 ± 17 mg/dL for the Control group and 388 ± 20 mg/dL for 6-OHDA group at min 30. However, this isolated alteration did not influence the area-under-curve (AUC) values, which revealed no changes in the overall glucose tolerance between the groups (Figure 6C). To verify whether motor impairment might be associated with an early change in insulin sensitivity, we performed ipITT on the 9th day of the experimental protocol. The insulin sensitivity remained unaltered in the 6-OHDA group compared with the Control group (Figures 6B,D). The absolute fed blood glucose values on baseline of ipITT were 118 ± 7 and 121 ± 8 mg/dL for the Control and 6-OHDA groups, respectively (*n* = 16–17, NS).

**FIGURE 7:**
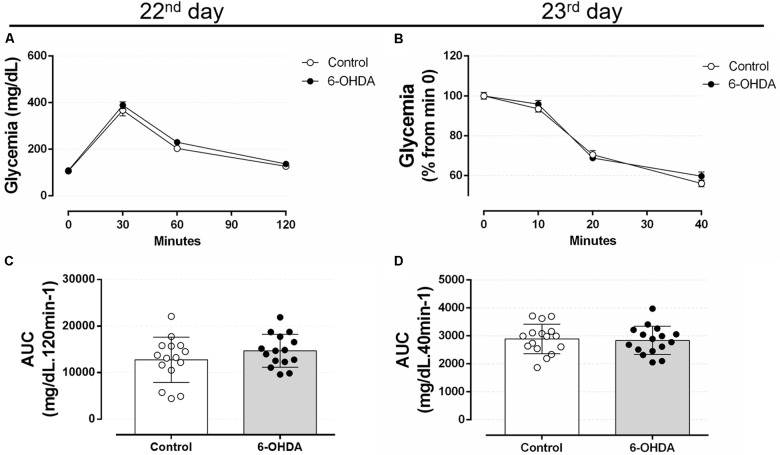
Blood glucose values during the intraperitoneal glucose and insulin tolerance test on the 22nd and 23rd day of the experimental protocol. **(A)** Blood glucose values during the ipGTT on the 22nd day. **(B)** Blood glucose values (normalized from min 0) during the ipITT on the 23rd day. **(C)** Area-under-curve during ipGTT and **(D)** during ipITT. Data are mean ± SEM in **(A,B)**, and mean ± SD in **(C,D)** (SEM were applied only for aesthetic reasons). Ordinary two-way ANOVA with repeated measures was applied in **(A,B)**, whereas unpaired Student’s *t*-test was applied in **(C,D)**. *n* = 16–17, *p <* 0.05.

The GTT values obtained in the 22nd postoperative day revealed no differences between the 6-OHDA and Control groups at 30, 60, and 120 min after intraperitoneal glucose load (Figures 7A,C). In addition, insulin sensitivity remained unaltered in the 23rd postoperative day in the 6-OHDA group when compared with the Control group (Figures 7B,D). The absolute fed blood glucose values on baseline of ipITT were 114 ± 5 and 112 ± 7 mg/dL for the Control and 6-OHDA groups, respectively (*n* = 16–17, NS).

**FIGURE 8:**
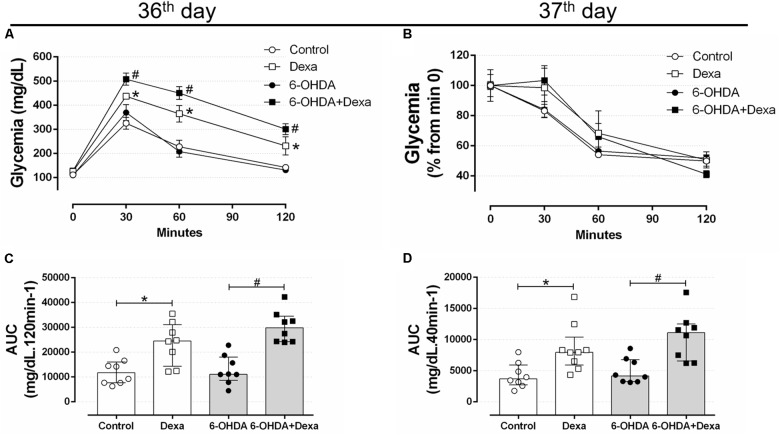
Blood glucose values during the intraperitoneal glucose and insulin tolerance test on the 36th and 37th day of the experimental protocol. **(A)** Blood glucose values during the ipGTT on the 36th day. **(B)** Blood glucose values (normalized from min 0) during the ipITT on the 37th day. **(C)** Area-under-curve during ipGTT and **(D)** during ipITT. Data are mean ± SEM in **(A,B)** (SEM were applied only for aesthetic reasons), and median and interquartile range as they are asymmetrically distributed (nonparametric) in **(C,D)**. ^∗^ and # Indicates a significant difference compared to the Control and 6-OHDA groups, respectively, using ordinary two-way ANOVA with repeated measures in **(A,B)**, or Kruskal–Wallis with Dunn’s *post hoc* test in **(C,D)**. *n* = 8–9, *p <* 0.05.

On the 36th day of the experimental protocol (5th day of dexamethasone treatment), treatment with GC resulted in glucose intolerance in the Dexa and 6-OHDA + Dexa groups when compared with the respective Control and 6-OHDA groups, as observed at 30, 60, and 120 min (Figure 8A) and in the AUC values (Figure 8C). Again, motor dysfunction caused by 6-OHDA did parallel with normal glucose tolerance even after 36 days postoperative and was associated with no exacerbation in the glucose intolerance caused by GC treatment. Glucose intolerance caused by dexamethasone treatment occurred parallelly to a significant decrease in the insulin sensitivity as observed by the ipITT data obtained on the 37th day (Figures 8B,D). 6-OHDA administration did not affect insulin sensitivity *per se* or exacerbated the action of GC treatment. The absolute fed blood glucose values on baseline of ipITT were 116 ± 5, 119 ± 7, 155 ± 39, and 180 ± 34 mg/dL for the Control, 6-OHDA, Dexa and 6-OHDA + Dexa groups, respectively (*n* = 8–9, *p* < 0.05 for dexamethasone-treated rats when compared with their respective 6-OHDA groups; only dexamethasone effect).

#### Central Administration of 6-OHDA Did Not Affect Plasma Insulin, Triacylglycerol, and Total Cholesterol Levels

**FIGURE 9:**
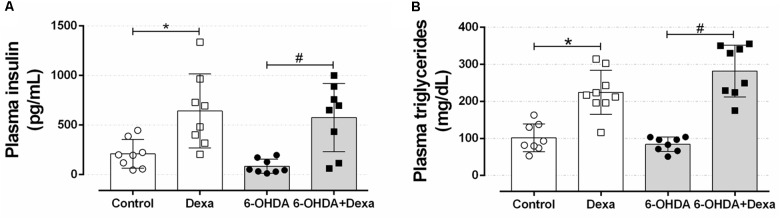
Circulating parameters. **(A)** Plasma insulin and **(B)** triacylglycerol concentration at the day of euthanasia on the 38th day of the experimental protocol. Data are median and interquartile range as they are asymmetrically distributed (nonparametric). ^∗^ and # Indicates a significant difference compared to the respective Control and 6-OHDA groups using Kruskal–Wallis with Dunn’s *post hoc* test. *n* = 8–9, *p <* 0.05.

To verify whether motor impairment caused by bilateral 6-OHDA administration could be associated with alteration in peripheral glucose and lipid metabolism, we evaluated the insulinemia and some circulating lipid levels. As observed in Figures 9A,B, rats receiving dexamethasone (Dexa and 6-OHDA + Dexa groups) exhibited an increase in plasma insulin levels compared with their respective control groups due to GC effect. Motor dysfunction induced by 6-OHDA was not associated with changes in insulinemia compared to the Control group (Figure 9A) and did not exacerbate the elevation in insulinemia caused by GC. In a very similar fashion, groups treated with dexamethasone showed an increase in plasma triacylglycerol levels, compared to their respective control groups (Control and 6-OHDA groups) (Figure 9B). No influence of 6-OHDA treatment *per se* was observed on this parameter. In addition, the four experimental groups evaluated did not show differences in plasma cholesterol levels (118, 108, 110, and 105 mg/dL for Control, Dexa, 6-OHDA and 6-OHDA + Dexa groups, respectively).

#### Central Administration of 6-OHDA Did Not Alter Hepatic Lipid and Glycogen Contents

**FIGURE 10:**
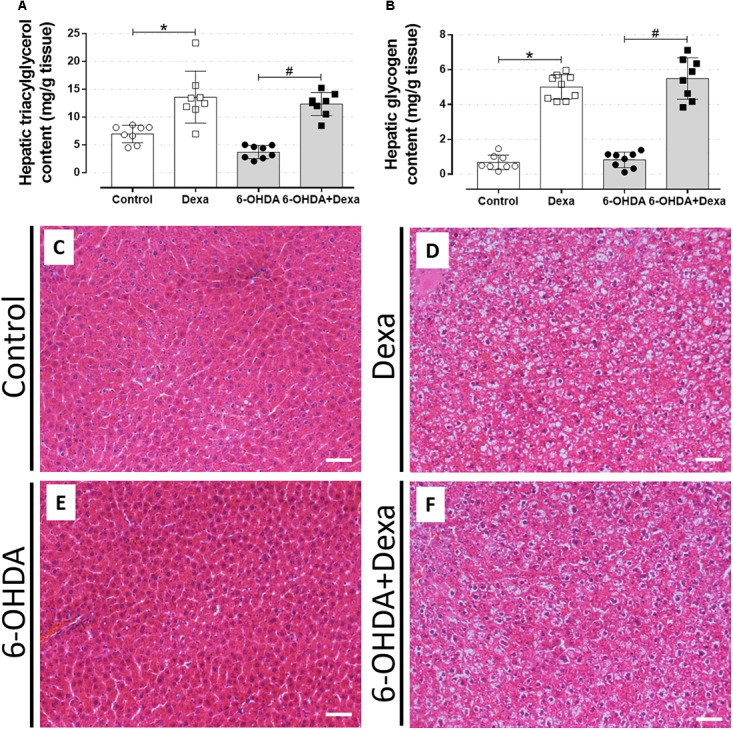
Liver parameters. **(A)** Hepatic triacylglycerol and **(B)** glycogen content at the day of euthanasia on the 38th day of the experimental protocol. Data are mean ± SD. ^∗^ and ^#^ indicate a significant difference compared to the respective Control and 6-OHDA groups using ordinary two-way ANOVA with Tukey’s *post hoc* test. *n* = 8–9, *p <* 0.05. **(C–F)** Representative liver sections from all groups in 200x magnitude stained with Hematoxylin and Eosin. *n* = 8–9. Scale bar in **(C–F)** represents 25 μm.

The accumulation of ectopic lipids is a common outcome in the contexts of metabolic diseases. Thus, to verify whether treatment with 6-OHDA influenced these parameters, we collected hepatic fragments to perform biochemical and histological analyses. As illustrated in Figures 10A,B, central administration of 6-OHDA did not influence the content of triacylglycerol and glycogen in the liver, respectively. GC-treated groups (Dexa and 6-OHDA + Dexa groups) showed higher hepatic triacylglycerol and glycogen contents when compared with the Control and 6-OHDA groups.

The morphological analysis of the liver sections revealed a significant phenotype of glycogen deposition and microvesicle lipids distribution in both the dexamethasone-treated groups (Table 1 and Figures 10D,F). Administration of 6-OHDA did not impact on lipid and glycogen content (Table 1 and Figure 10E). As expected, none of the liver sections from the Control group exhibited signs of glycogen and triacylglycerol contents (Table 1 and Figure 10C).

**Table 1:** An indication of the presence of lipid and/or glycogen deposition.

	Lipids	Glycogen
Control	0/8	0/8
Dexa	8/9^∗^	8/9^∗^
6-OHDA	1/8	1/8
6-OHDA+Dexa	8/8^#^	8/8^#^

*Numbers indicate the presence of phenotype per total number of sections analyzed. Each section represents a different rat. ^∗^ and ^#^ indicates a significant difference compared to the respective Control and 6-OHDA groups, respectively using Pearson Chi-square test, p < 0.05, n = 8–9.*

#### Central Administration of 6-OHDA Did Not Alter Adiposity and Metabolic Organ Masses

After perfusion and euthanasia of the animals, the relative mass of omental, epididymal, and retroperitoneal fat depots and metabolic organs were quantified. Neither 6-OHDA nor GC administration altered the visceral adiposity as observed in Table 2. Liver and spleen masses remained unaltered too. It was only observed a reduction in the adrenal gland mass in the 6-OHDA + Dexa group compared to the 6-OHDA group.

**Table 2:** Organ masses (g/100 g body mass) on the day of euthanasia.

	Control	Dexa	6-OHDA	6-OHDA + Dexa
Omental fat	0.17[0.12;0.23]	0.17[0.16;0.24]	0.15[0.11;0.19]	0.16[0.09;0.24]
Epididymal fat	1.67[1.51;1.76]	1.85[1.48;1.87]	1.42[1.35;1.57]	1.68[1.18;2.25]
Retroperitoneal fat	1.58[1.43;2.12]	1.32[1.16;1.70]	1.38[1.12;1.53]	1.51[1.11;1.88]
Liver	4.15[3.83; 4.49]	4.92[4.24;5.05]	4.19[3.25;4.35]	4.46[4.11;4.93]
Spleen	0.18[0.16;0.19]	0.15[0.13;0.17]	0.18[0.17;0.19]	0.14[0.13;0.17]
Adrenals	0.02[0.01;0.02]	0.01[0.01;0.01]	0.02[0.01;0.02]	0.01[0.01;0.01]#

*Results are expressed as a median ± interquartile range. The (#) indicates a significant difference compared to the 6-OHDA group using Kruskal–Wallis with Dunn’s post hoc test. (n = 8, p < 0.05; see main text for a detailed description).*

#### Central Administration of 6-OHDA Did Not Impact on Endocrine Pancreas Mass

**FIGURE 11:**
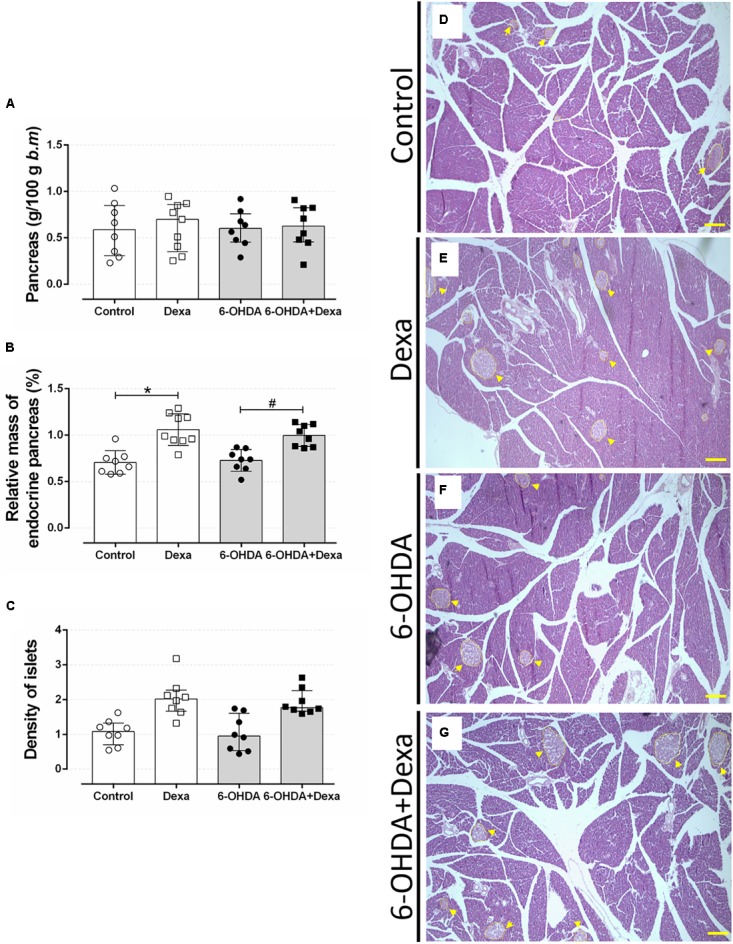
Mass of endocrine pancreas. **(A)** The relative pancreas mass, **(B)** relative pancreatic islet mass, and **(C)** the islet density (islet number per 1000 μm^2^ pancreas section) at the day of euthanasia on the 38th day of the experimental protocol. Data are mean ± SD. ^∗^ and # Indicate a significant difference compared to the respective Control and 6-OHDA groups using ordinary two-way ANOVA with Tukey’s *post hoc* test. *n* = 8–9, *p <* 0.05. **(D–G)** representative pancreas sections from all groups in 40x magnitude stained with Hematoxylin and Eosin. *n* = 8–9. Scale bar in **(D–G)** represents 200 μm. Yellow arrowheads are pointing to pancreatic islets.

Increase in the endocrine pancreas mass is an adaptive compensatory response to a reduction in peripheral insulin sensitivity. Thus, we evaluated whether motor dysfunction could be associated with the impairment of such expected adaptation in GC-treated rats. No differences were observed in the relative mass of the pancreas between the four groups (Figure 11A). The increase in the relative pancreatic islet mass caused by GC treatment was not affected by 6-OHDA treatment (Figures 11B,D–G). The increase of the relative mass was accompanied by a no significant increase of islet density (number of islets per 1000 μm^2^ of pancreas section) in the four experimental groups (Figure 11C).

## Discussion

In the present study, we investigated the impact of bilateral administration of 6-OHDA on the striatum of rats on metabolic parameters to verify whether a persistent motor dysfunction could affect the glucose and lipid homeostasis in a later period. Overall, we observed that central administration of 6-OHDA reproduced the motor dysfunction (i.e., impairment of locomotion and motor coordination) as evidenced in the open field and rotarod tests, which was persistent for the entire period of the experiment. This motor dysfunction was not associated with alteration of glucose tolerance and insulin sensitivity or circulating lipids levels and did not exacerbate the classical adverse effects of excessive GCs.

To achieve a persistent motor dysfunction, we selected the PD rat model of bilateral 6-OHDA administration (Sauer and Oertel, 1994; Przedborski et al., 1995; Lee et al., 1996). With this approach occurs a neuronal lesion that gradually evolves from the nigrostriatal pathway, 1 day after the stereotaxic procedure, to the nigral cells, 1 week after injection, reaching the maximum degeneration between 2 and 3 weeks (Sauer and Oertel, 1994; Przedborski et al., 1995; Blandini et al., 2007). In addition, the dose of 20 μg administered in the striatum promotes a loss of dopaminergic cells varying between 20 and 85% in the SNpc region and 60–90% in the striatum region up to 2 weeks after the surgical procedure (Przedborski et al., 1995; Lee et al., 1996; Kirik et al., 1998; Blandini et al., 2007).

In human PD there is an extensive loss of dopaminergic neurons from the SNpc, resulting in a decline in dopamine concentrations of the striatum, which results in several motor deficiencies including rigidity, tremor, and loss of postural reflexes (Riederer and Wuketich, 1976; Beal, 2005). In *in vivo* studies with rodents, dopaminergic loss resulting from the action of the 6-OHDA toxin and confirmation of motor impairments can be detected through analyses of motor behaviors. In our study, infusion of 6-OHDA into the dorsolateral striatum of rats resulted in motor impairment such as decreased locomotor activity at 7 and 35 days after 6-OHDA administration (Figures 2, 3). These results are supported by numerous previous studies. Bilateral administration of 20 μg of 6-OHDA in the dorsolateral striatum of rats promotes significant deficits in locomotor activity addressed in the open field test, 21 days after 6-OHDA administration (Matheus et al., 2016). Similarly, motor impairments were observed with unilateral administration of 10 μg of 6-OHDA in the striatum, which included a decrease in locomotion and exploratory behavior of male Wistar rats, observed 3 weeks after treatment with the 6-OHDA neurotoxin (Hegazy et al., 2017). Locomotor and exploratory deficits can also be achieved with bilateral 6-OHDA administration of 15 μg in the striatum of rats (Rampersaud et al., 2012).

In the present study, the 6-OHDA-treated rats also exhibited impaired motor coordination (Figure 4). This motor dysfunction is supported by several studies. For instance, the motor coordination was impaired after 1 week of unilateral 6-OHDA administration of 28 μg or bilateral 6-OHDA administration of 20 μg in rats (Monville et al., 2006; Kumar et al., 2012). These findings support our results demonstrating the validity of our experimental animal model. It is important to emphasize that 6-OHDA-lesioned rats displayed motor dysfunctions for the entire experimental protocol duration in the present study, corroborating findings from previous studies (Rampersaud et al., 2012; Matheus et al., 2016).

Although we were not searching for the impact of GC treatment on central outcomes, there is evidence suggesting that 7 days of treatment with dexamethasone (1 mg/kg b.m.) in 6-OHDA-treated mice may partially inhibit microglial activation by attenuating the production of proinflammatory cytokines (Kurkowska-Jastrzebska et al., 2004). This anti-inflammatory action of GCs could attenuate the degeneration of dopaminergic neurons in *in vivo* models of PD improving some motor deficits. In our study, no apparent attenuation that could be attributable to GCs were observed in the behavioral tests performed on the 35th day of the experimental protocol (4th day of exposure to GC). This apparent discrepancy can be explained, at least in part, because in this latter study with mouse the GC was administered previously to 6-OHDA lesion.

Murinometric data revealed no impact of 6-OHDA administration on body mass and on food intake in the present study (Figure 5). The nigrostriatal dopaminergic system plays an essential role in the regulation of appetite and body weight (Ungerstedt, 1971; Fibiger et al., 1973; Lénárd, 1977). In addition, there is evidence in mouse showing that hypothalamic arcuate nucleus (ARC) possesses TH-positive neurons and that these neurons play an orexigenic role in energy homeostasis (Zhang and van den Pol, 2016). Evidence obtained in animal models suggests that dopaminergic lesions greater than 95% induce adipsia and hypophagia (Ungerstedt, 1971; Sakai and Gash, 1994). However, in the present study, the animals were submitted to a lesion in the striatum that is expected to produce a slower progression of motor symptoms, which may have prevented any relevant influence on the control of food intake. Studies have shown that patients with PD tend to lose weight with disease progression, presenting a lower body weight when compared to the corresponding age-control population. Explanations for weight loss of PD patients include difficulty in chewing and swallowing, increased time required to eat, decreased olfactory and palatability, depression, and increased energy requirements intake due to muscular rigidity and involuntary movements (Beyer et al., 1995; Lorefält et al., 2006). Whether neurodegeneration in dopaminergic neurons outside striatum (i.e., hypothalamic ARC) in PD are also affected, and how much this can influence the metabolism from PD patients, is a question that merit investigation. Dayan et al. (2018) demonstrated that PD patients with symptoms of autonomic impairments exhibit disrupted thalamic-striatal-hypothalamic functional connectivity, suggesting the possible involvement of neural circuits that regulate autonomic function. Reduction in both body mass and food intake is a well-known adverse effect of high GC doses, as previously demonstrated in rats (Giozzet et al., 2008; dos Santos et al., 2014; Protzek et al., 2014). Numerous mechanisms by which GCs act on body mass and appetite includes: (i) suppression of osteoblastogenesis, as well as apoptosis of osteoblasts, decreasing bone density (Manolagas and Weinstein, 1999); (ii) increase of lipolysis in adipose tissue and catabolic effect on muscle tissue (Franco-Colin et al., 2000); (iii) decrease of expression of the orexigenic peptides NPY and AgRP (Liu et al., 2011); (iv) increase of circulating anorexigenic hormones insulin and leptin (Caldefie-Chézet et al., 2001; Giozzet et al., 2008; dos Santos et al., 2014; Protzek et al., 2014); and (v) by a negative water balance (Thunhorst et al., 2007).

Glucose tolerance and insulin sensitivity remained unaltered during the entire experimental protocol, despite the presence of consistent motor dysfunction caused by 6-OHDA administration (Figures 6–8). Some studies suggested the possible association between PD and impairment in insulin signaling. A study with rats submitted to the unilateral 6-OHDA administration of 12 μg in SNpc demonstrated an increase in serine phosphorylation of the insulin receptor substrate 2 that parallels with a depletion of dopamine (White, 2002; Morris et al., 2008). However, functional parameters reflecting the balance of glucose metabolism were not investigated by the authors. The presence of motor dysfunction caused by 6-OHDA did not exacerbate the known adverse effects of GC on glucose tolerance and insulin sensitivity in our study (Figures 6–8). The negative action of GC on glucose tolerance and in peripheral insulin sensitivity has been described in several clinical and preclinical studies (for a detailed review, refers to Pasieka and Rafacho, 2016). GCs treatment may alter glucose homeostasis through elevation of hepatic glucose output, reduction of skeletal muscle glucose uptake and an increase of adipose tissue lipolysis (Pasieka and Rafacho, 2016). Decreased insulin sensitivity is commonly accompanied by an increase in plasma insulin concentrations and an increase in the relative mass of pancreatic islets (Kahn et al., 1993), events observed in our GC-treated rats (Figures 9, 11). Although we did not evaluate the *in vivo* insulin secretion under ipGTT experiment, the suggestive insulin hypersecretion observed by our hyperinsulinemic rats was not enough to prevent glucose intolerance and this is supported by previous studies (Giozzet et al., 2008; Angelini et al., 2010; Protzek et al., 2014; dos Santos et al., 2014), an effect that was not exacerbated in 6-OHDA-treated rats.

The impairment in glucose homeostasis may be followed by an imbalance in lipid homeostasis. Consistent with the diabetogenic effects of GCs, dexamethasone-treated rats had increased levels of circulating triacylglycerides, with a parallel increase in hepatic fat content (Figures 9B, 10A respectively). There is evidence demonstrating that GCs may increase circulating and hepatic triacylglycerol levels by inhibiting lipoprotein lipase in adipose tissue (Franco-Colin et al., 2000) and upregulating hepatic lipogenic enzymes (Motta et al., 2018). The presence of motor dysfunctions seemed to be innocuous on these lipidic measures. The relative masses of metabolic organs, except for the hypotrophy of adrenal gland mass in Dexa + 6-OHDA group, were unaltered and the perfusion process may have masked the visualization of these changes since these parenchymas retained fixative solution used for the perfusion procedure.

One of the limitations of our study resides in the fact that we did not appreciate the TH distribution or quantification by immunohistochemistry or western blot experiments, respectively. However, there is evidence for a sustained lesion in the striatum and in the SNpc up to 8 weeks after unilateral (Perlbarg et al., 2018) or bilateral (Oliveira et al., 2017; Fernandes-Junior et al., 2018) administration of 6-OHDA in rats. No motor function parameters were evaluated in these aforementioned studies demonstrating that not all studies include both analyses (motor analysis and TH immunostaining) to confirm the effectiveness of 6-OHDA administration.

The relationship between neurodegeneration and metabolic outcomes are complex and is difficult to predict which comes first. This crosstalk is influenced by numerous factors, which turn difficult to precisely define the causal factor(s). For instance, there is evidence linking the severity of neurodegeneration caused by 6-OHDA with a previous metabolic status (i.e., diet-induced obesity) in rats (Morris et al., 2010). Similarly, there is also evidence linking the inflammation of hypothalamus triggered by Aβ oligomers infusion with posterior disruption of metabolic homeostasis (Clarke et al., 2015), suggesting a relationship between central and peripheral components. With our current experimental design, we defined the independent factor (treatment with 6-OHDA) and then considered all metabolic outcomes as dependent variables, and by doing this we tried to isolate as much as possible the causal factor.

Recent evidence demonstrated the relationship between metabolic disorders and consequent negative prognostic for PD. For instance, coexistence of T2DM with PD lead to a faster motor progression and cognitive decline (Pagano et al., 2018) as well as reduction in the baseline dopamine transport availability in the caudate and ventral striatum, cortical thinning in the inferomedial temporal lobe that is also associated with reduced cognitive performances (Chung et al., 2018) when compared to patient with only PD. In addition, the content of the tau and α-synuclein in the cerebrospinal fluid is higher in patients with both PD and T2DM in relation to patients only with PD, and these markers are already upregulated in T2DM patients compared with healthy controls (Pagano et al., 2018), indicating a relationship between PD-pathology markers and T2DM. This mutual relationship is reinforced when considering the benefits of some antidiabetic drugs (i.e., agonists of glucagon-like peptide-1 receptor and inhibitors of dipeptidyl peptidase-4) exerts on the neurodegenerative process in patients with PD (Ashraghi et al., 2016).

A recent study conducted by Ter Horst et al. (2018) revealed that bilateral deep brain stimulation targeting the border of the nucleus accumbens (NAc) core and ventral anterior limb of the internal capsule ameliorates some metabolic aspects of patients with diabetes (i.e., improves the peripheral insulin sensitivity). This study also demonstrated that pharmacological dopamine deletion decreases insulin sensitivity, whereas optogenetic activation of NAc dopaminergic-receptor-1 neurons improves glucose tolerance in mice, suggesting that striatal dopamine signaling plays a role in glucose metabolism.

## Conclusion

Our findings let us conclude that bilateral 6-OHDA administration in the dorsolateral striatum causes sustained motor impairments with no impact on glucose and lipid homeostasis and does not exacerbate the adverse effects caused by excess GC in glucose and lipid metabolism. These observations suggest that on this two-way road between PD and peripheral metabolism, it is probable that dopaminergic circuit dysfunction has a minor impact on metabolic outcomes.

## Data Availability Statement

The datasets generated to support the findings of this study are available from the corresponding author upon reasonable request.

## Author Contributions

AR, EM, FG, and RP contributed to the experimental design. FG, FdS, MB, and RF conducted the experiments. AR and FG contributed with analytic tools and data analysis, performed the data collection, and wrote up the manuscript. AR, DZ, and RP supplied reagents and materials. AR, EM, and FG contributed to the discussion of the experimental findings. All authors read and approved the manuscript’s final format.

## Conflict of Interest Statement

The authors declare that the research was conducted in the absence of any commercial or financial relationships that could be construed as a potential conflict of interest.
